# Rare Radiographic Changes in a Cervicomedullary Spinal Ependymoma After Biopsy and Drainage of an Intratumoral Cyst: A Staged Approach

**DOI:** 10.7759/cureus.34017

**Published:** 2023-01-20

**Authors:** Catherine E Wassef, Melissa R Holloway, Howard Silberstein

**Affiliations:** 1 Neurosurgery, University of Rochester Medical Center, Rochester, USA

**Keywords:** spinal ependymoma, spinal cord tumor surgery, intramedullary spinal cord tumor, intratumoral cyst, spinal fusion

## Abstract

Spinal ependymomas are the most common intramedullary spinal tumor, with a large proportion containing a small intratumoral cyst. Although the signal intensity varies, spinal ependymomas are generally well-demarcated, are not associated with a pre-syrinx, and do not extend above the foramen magnum. Our case demonstrates unique radiographic findings of a cervical ependymoma with a staged approach to diagnosis and resection. The patient is a 19-year-old female who presented with a three-year history of neck pain, progressive arm and leg weakness, falls, and functional decline. MRI revealed an expansile dorsal and centrally located T2 hypointense cervical lesion with a large intratumoral cyst extending from the foramen magnum to the C7 pedicle. Contrasted T1 scans showed an irregular enhancement pattern along the superior tumoral border down to the C3 pedicle. She underwent a C1 laminectomy for open biopsy and cysto-subarachnoid shunt. Postoperative MRI revealed a well-demarcated enhancing mass extending from the foramen magnum to C2. Pathology revealed Grade II ependymoma. She underwent an occipital to C3 laminectomy with gross total resection. Postoperatively she experienced weakness and orthostatic hypotension that improved remarkably upon discharge. Initial imaging was concerning for a higher-grade tumor, with holocervical cord involvement and cervical kyphosis. Given concern for grade and possible extensive C1-7 laminectomy and fusion for resection, a smaller surgery involving drainage of the cyst and biopsy was performed. Postoperative MRI revealed regression of the pre-syrinx, improved tumoral definition, and improvement of cervical kyphosis. This staged approach spared the patient unnecessary surgical intervention such as extensive laminectomy and fusion. We conclude that in cases of a large intratumoral cyst in an extensive intramedullary spinal cord lesion, open biopsy and drainage followed by resection in a staged fashion should be considered. Radiographic changes from the first procedure may affect the surgical approach for ultimate resection.

## Introduction

Spinal ependymomas (WHO grade II) are the most common intramedullary spinal tumors, comprising approximately 60% of glial spinal cord tumors in adults and 30% in pediatric patients [1]. Almost all tumors are located centrally, though recent evidence suggests that these tumors arise from progenitor cells or radial glia-like cells, rather than from the central canal as originally thought [2]. The cervical spinal cord is the most common site of ependymoma involvement, with tumors rarely extending above the foramen magnum. Classic presenting symptoms are generally slowly progressive and include pain, weakness, sensory changes, and motor and autonomic dysfunction [3].

Classic grade II spinal ependymomas, also referred to as the cellular type, are generally well demarcated and accepted to exhibit classic imaging patterns, including iso- to hypo-intensity on T1-weighted MRI, hyperintensity on T2-weighted MRI, and strong homogenous gadolinium (Gd) enhancement on contrasted images [4]. However, common pathological features of ependymomas can interfere with these imaging patterns, obscuring expected findings. These features classically include associated cysts, syringomyelia, and a hypointense hemosiderin “cap sign” suggestive of hemorrhage [5].

The majority of spinal ependymomas are associated with a cystic component, which can be divided into the following three distinct types: intratumoral cysts (ITCs), rostral or caudal (polar or satellite) cysts, and reactive dilation of the central canal (syringomyelia) [6]. Early research indicates that polar cysts are more common than ITCs, though recent evidence suggests that polar cysts may simply form from ITCs that migrated after outgrowing their vascular supply, and low ITC estimates may be a product of underdiagnosis [3]. Current literature estimates that 22-84% of spinal ependymomas contain ITCs [7].

Cystic involvement is important to recognize early as cystic presence may alter MRI interpretation, cyst volume has documented proportionality to symptom duration and severity, and cyst size and location may affect recommended treatment [4]. Classically, gross total resection (GTR) is the gold standard of treatment for Grade II spinal ependymomas, with resection of ITCs and drainage of polar cysts intraoperatively [3]. To our knowledge, ours is the first documented case in the literature in which a staged approach to ITC drainage and subsequent tumor resection was employed.

This article was previously presented as a poster at the 2022 AACNS Annual Congress on September 6, 2022.

## Case presentation

**Figure 1:**
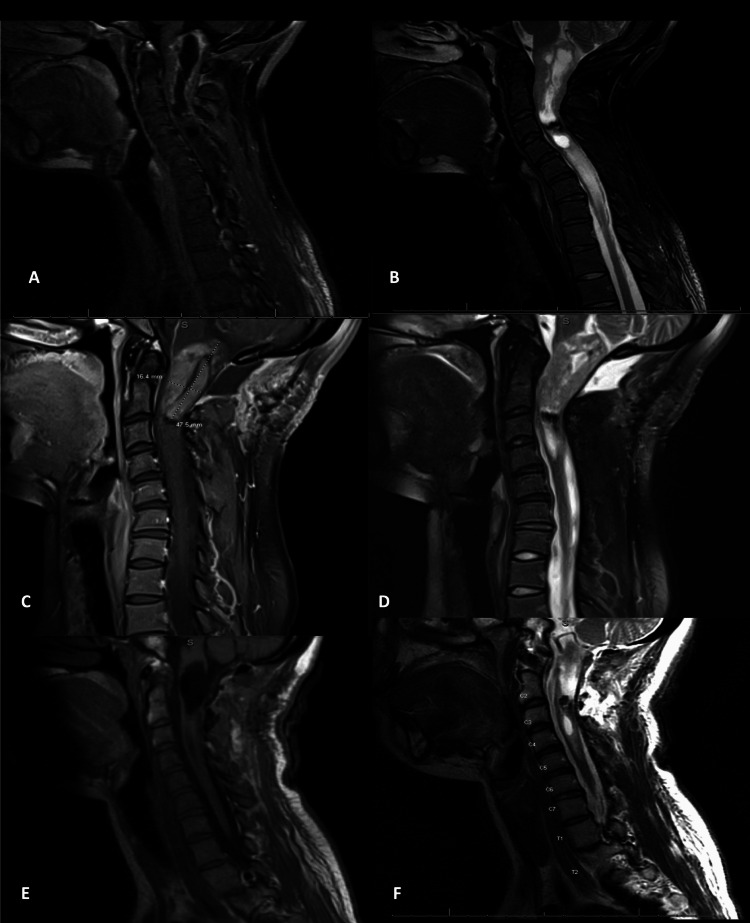
Sagittal magnetic resonance imaging findings. (A) Preoperative T1 image with contrast. (B) Preoperative T2 image. (C) Post-biopsy and drainage T1 image with contrast. (D) Post-biopsy and drainage T2 image. (E) Post-resection T1 image with contrast. (F) Post-resection T2 image.

**Figure 2:**
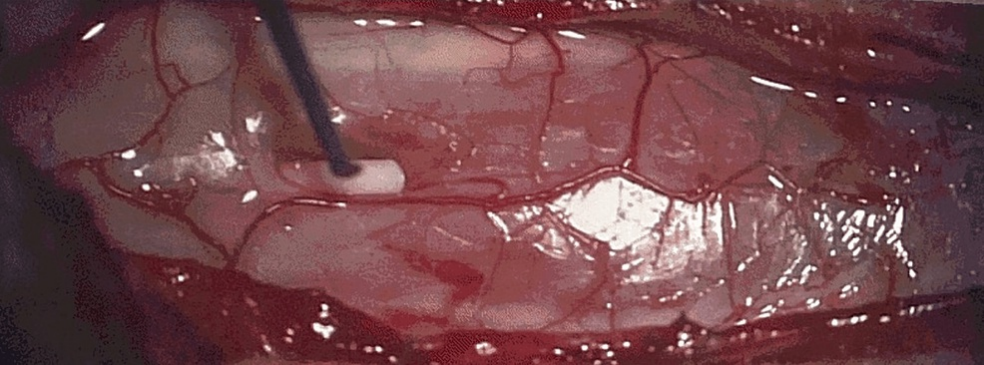
Intraoperative image. Intraoperative photograph at second-stage resection. White tubing in the center of the canal is the syringo-subarachnoid shunt tube. Normal spinal cord and dorsal columns are seen as white linear structures along either side of the dark pink mass below the pial surface, which is ependymoma.

A 19-year-old female with a past medical history of congenital hearing loss presented with a three-year history of neck pain, progressive arm and leg weakness, falls, and functional decline. She had developed myelopathic gait and bilateral upper extremity weakness, which was very prominent in the hands. MRI revealed an expansile dorsal and centrally located T2 hypointense cervical lesion with a large ITC extending from the foramen magnum to the C7 pedicle. T2 central hypodensity extended beyond the cyst to the level of the T1-2 disc space, concerning for either infiltrative tumor, pre-syrinx, or syrinx with surrounding edema (Figures 1A, 1B). Contrasted T1 scans showed an irregular enhancement pattern along the superior tumoral border down to the C3 pedicle. Lastly, she had some reversal of cervical lordosis at C5-6 with the maintenance of overall cervical lordosis. A multidisciplinary meeting between pediatric neurosurgery, neuroradiology, neuro-oncology, and radiation oncology concluded that this tumor was likely of higher grade and difficult to resect, but that tissue diagnosis was necessary to proceed with any adjuvant therapy. She underwent a C1 laminectomy for open biopsy and cysto-subarachnoid shunt, which can be seen in the second-stage intraoperative photograph in Figure 2. Her weakness subjectively improved significantly. Postoperative MRI revealed a well-demarcated more homogenously enhancing mass extending from the foramen magnum to C2 in addition to a caudal polar cyst with a resolution of pre-syrinx edema (Figures 1C, 1D). Pathology revealed Grade II ependymoma. She subsequently underwent an occipital to C3 laminectomy with GTR. As seen in Figure 2, the tumor borders appear well-defined. Post-resection, no residual enhancement was seen, and the hemosiderin cap was seen centrally adjacent to the C2-3 disc space (Figures 1E, 1F). Postoperatively, she experienced weakness and orthostatic hypotension that improved remarkably upon discharge. The orthostatic hypotension was treated with pseudoephedrine. At a three-month outpatient follow-up, the patient was still suffering from episodic orthostatic hypotension, which was continuing to improve. Her arm strength and function continued to improve as well. She reported some neck soreness, and MRI revealed similar C5-6 kyphosis when compared to preoperative MRI, for which she was referred for physical therapy. She also underwent adjuvant radiation of 50.4 Gray administered to the tumor bed in 28 fractions. Chemotherapy was deferred, and she will continue to undergo surveillance imaging of her tumor and cervical kyphosis.

## Discussion

Several operative considerations were relevant to this case. First, although GTR is the traditional treatment gold standard for spinal ependymomas, this outcome is not possible in the case of malignant tumors, and in benign cases, the potential benefit must be weighed carefully against the preoperative clinical deficits, as almost half of patients experience worsening of their long-term neurological status postoperatively [3]. Second, initial concern for a holocervical tumor would prompt consideration for staged resection, as this approach is beneficial in patients with holocord ependymomas or with large, complex body habitus which may complicate the surgery. In these patients, cervical and thoracic segments may be resected in a staged fashion. Tumors confined to the cervical segment can generally be resected during the same surgery [8]. In our patient, resection of a holocervical tumor would have required a C1-7 laminectomy and most likely fusion surgery. One study revealed that in a group of patients undergoing intradural spinal tumor resection, for each increasing number of laminae removed, there was a 3.1-fold increase in the likelihood of instability requiring cervical fusion, and those patients who underwent more than three-level laminectomy were 9.5 times more likely to require cervical fusion than those in whom less than three levels were treated [9]. This, again, requires careful consideration of the preoperative deficits and clinical course, as cervical fusion surgery is associated with serious risk and morbidity, especially in younger patients, with adjacent segment degeneration present in up to one-third of patients postoperatively [10]. In the case of benign tumors, there may be a benefit in watching the patient clinically in lieu of surgery.

Employing a staged approach to cyst drainage and tumor resection with this patient yielded more accurate diagnostic information, allowing our team to make informed treatment decisions. This method revealed that a lesion which, on initial imaging, was concerning for a high-grade tumor with holocervical cord involvement and cervical kyphosis, spanned only from C1 to C3. The patient was spared unnecessary surgical intervention such as extensive laminectomy and fusion, and GTR was achieved. Postoperatively, chemotherapy and radiation are not recommended for this patient, as these treatments are usually reserved for incomplete resections and higher grade (WHO grade III-IV) tumors [11,12].

## Conclusions

In cases of large ITC in an extensive intramedullary spinal lesion, precise accurate imaging may be disrupted by the cyst, disruption in cerebrospinal circulation, and associated features including hemosiderin cap. Diffuse cord signal change in these tumors may be mistaken for tumor but pre-syrinx and peritumoral edema should also be considered. Open biopsy and drainage followed by resection in a staged fashion should be considered in these patients, as large, malignant appearing intramedullary tumors with extensive ITCs may appear lower grade after cyst drainage. Radiographic changes from the first procedure may affect the surgical approach for ultimate resection.
